# Dealing with an Unconventional Genetic Code in Mitochondria: The Biogenesis and Pathogenic Defects of the 5-Formylcytosine Modification in Mitochondrial tRNA^Met^

**DOI:** 10.3390/biom7010024

**Published:** 2017-03-02

**Authors:** Lindsey Van Haute, Christopher A. Powell, Michal Minczuk

**Affiliations:** Mitochondrial Genetics, Medical Research Council Mitochondrial Biology Unit, University of Cambridge, Wellcome Trust/MRC Building, Cambridge Biomedical Campus, Hills Road, Cambridge CB2 0XY, UK; cap71@mrc-mbu.cam.ac.uk

**Keywords:** mitochondria, tRNA, NSUN3, 5-methylcytosine, 5-formylcytosine, RNA modification, translation

## Abstract

Human mitochondria contain their own genome, which uses an unconventional genetic code. In addition to the standard AUG methionine codon, the single mitochondrial tRNA Methionine (mt-tRNA^Met^) also recognises AUA during translation initiation and elongation. Post-transcriptional modifications of tRNAs are important for structure, stability, correct folding and aminoacylation as well as decoding. The unique 5-formylcytosine (f^5^C) modification of position 34 in mt-tRNA^Met^ has been long postulated to be crucial for decoding of unconventional methionine codons and efficient mitochondrial translation. However, the enzymes responsible for the formation of mitochondrial f^5^C have been identified only recently. The first step of the f^5^C pathway consists of methylation of cytosine by NSUN3. This is followed by further oxidation by ABH1. Here, we review the role of f^5^C, the latest breakthroughs in our understanding of the biogenesis of this unique mitochondrial tRNA modification and its involvement in human disease.

## 1. Introduction

Mitochondria have their own DNA (mtDNA) that encodes thirteen essential subunits of the oxidative phosphorylation (OXPHOS) system. Apart from these genes, the human mitochondrial transcriptome also consists of two ribosomal RNAs (mt-rRNA) and a full set of 22 transfer RNAs (mt-tRNA). All other proteins necessary for the expression of mtDNA, including those responsible for post-transcriptional RNA modifications, are encoded by nuclear genes (nDNA) and imported into mitochondria upon translation in the cytosol. Perturbation of mitochondrial gene expression can lead mitochondrial diseases. The pathological defects of mitochondrial gene expression can result from mutations either in mtDNA or nDNA. For a broad overview of processes and proteins involved in mitochondrial gene expression and their role in human pathology, we refer to recent reviews [1,2,3,4,5,6,7].

The translation of a messenger RNA (mRNA) into its corresponding polypeptide chain is dependent on the precise interactions between the three bases of the mRNA’s triplet codon and the triplet anticodon of the cognate tRNA. In mammalian mitochondria, all mt-tRNAs have to recognize at least two different codons. For any given mt-tRNA, the recognized codons always share the same base identity at the first and second positions, but differ at the third. Consequently, position 34 (the first position of the anticodon, the “wobble base”) in the mt-tRNA cannot always base pair with the third nucleotide of the codon according to the conventional Watson–Crick pairing rules. There are eight mt-tRNAs that recognize four codons each. In all these cases, position 34 of the mt-tRNA, the wobble base, is occupied by uridine, which is capable of base pairing with any of the four bases due to enhanced conformational flexibility within the anticodon loop [8]. However, the remaining fourteen tRNAs interact with a purine or a pyrimidine in the third codon position and consequently recognize exactly two codons. This increase in discrimination by the wobble base is achieved through post-transcriptional modifications.

The mammalian mitochondrial genetic code differs from the universal genetic code by using unconventional codons [9]. UGA encodes tryptophan. AGA and AGG (AGR), which encode arginine in the universal code, are not used by mitochondrial open reading frames (ORFs) during translation elongation and have been for years recognized as ‘stop’ signals. However, the use of the AGR codons as stop signal has been questioned by the observation of a -1 frameshift by the mitoribosome, which places a standard UAG stop codon at the A-site [10], and is still the matter of lively debate [11,12,13]. Finally, in addition to conventional AUG, methionine coding is expanded to AUA (as well as AUU, but only as an initiation codon), with all these codons being recognized by a single tRNA Methionine (tRNA^Met^) bearing a CAU anticodon, serving as both the elongator and initiator tRNA [14].

As with all known tRNAs, mt-tRNAs undergo numerous post-transcriptional nucleotide modifications and a great range of chemical diversity exists with bases undergoing methylations and formylations, along with several others [15,16]. Chemical nucleotide modifications are crucial for tRNA structure, stability, correct folding and aminoacylation. Additionally, modifications ensure the efficiency and stringent accuracy that is required during decoding in mitochondrial translation. Mitochondrial tRNA modifications are introduced by several site-specific enzymes encoded in the nucleus. For a detailed overview of known nuclear factors that are involved in post-transcriptional processing and modification of mt-tRNAs and their role in mitochondrial disease, we refer to other recently published work [15,17,18,19,20,21,22,23,24,25,26,27].

This review article focuses on the 5-formylcytosine (f^5^C) modification of the wobble position of mt-tRNA^Met^, which allows for the recognition of unconventional mitochondrial methionine codons. We also describe the recent discovery of a sequential pathway and the enzymes involved in the generation of this post-transcriptional modification. We discuss the in vitro characterization of this modification and its possible role in vivo. Furthermore, we describe the role of mutations in mt-tRNA^Met^, their effect on f^5^C formation and the lack of f^5^C in a patient with mitochondrial disease.

## 2. Discovery and Formation of 5-Formylcytosine at Position C34 of mt-tRNA^Met^

In 1994, a novel and unique modified nucleoside, f^5^C, was found at the wobble position of bovine liver mitochondrial tRNA^Met^ [28] and the parasitic nematode *Ascaris suum* [29]. In the subsequent years, it was shown that mt-tRNA^Met^ from *Loligo breekeri* (squid) [30], *Drosophila melanogaster* (fruit fly) [31], *Gallus domesticus* (chicken), *Xenopus laevis* (frog) and *Rattus norvegicus* (rat) [32] also possess f^5^C34 and it was therefore considered universal in eukaryotic mt-tRNA^Met^. However, the presence of this modification in human mt-tRNA^Met^ has only recently been confirmed [33,34]. f^5^C has not been detected in other mitochondrial RNAs.

**Figure 1:**
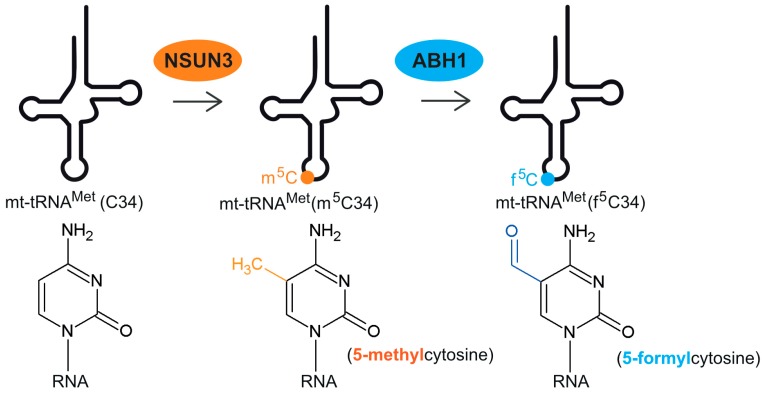
Graphical overview of the tRNA Methionine (mt-tRNA^Met^) formylation pathway. NSUN3 methylates unmodified C34 to form 5-methylcytosine (m^5^C) which is then further oxidized into 5-formylcytosine (f^5^C) by ABH1.

Although f^5^C34 on mt-tRNA^Met^ was identified almost three decades ago, the enzymes responsible for this modification have only very recently been discovered by three independent groups, almost simultaneously [33,34,35]. It was shown that initially a methyl group is added to the cytosine at position 34 of mt-tRNA^Met^, which is then further converted to a formyl group. One of the key experiments to reveal the biosynthetic pathway of f^5^C, was to identify the carbon source of the C34 formyl group of mt-tRNA^Met^. Many metabolites use formyl-tetrahydrofolate (formyl-THF) as the formyl donor. On the other hand, 5-formyldeoxycytidine (f^5^dC) found as a stable modification of DNA [36], is generated by oxidation of the 5-methyldeoxycytidine (m^5^dC) intermediate. In this case, the carbon from the methyl group donor, *S*-adenosyl methionine (SAM), is found in the f^5^dC formyl group. Metabolic isotope labelling with precursors of formyl-THF or SAM revealed that the carbon atom of the formyl group in mt-tRNA^Met^ f^5^C34 was derived from SAM rather than formyl-THF. These results suggested stepwise biogenesis of f^5^C34 with an initial SAM-dependent methylation of C34, to form m^5^C34, followed by hydroxylation and oxidation of the methyl group (Figure 1), reminiscent of m^5^dC formation in DNA [33].

The methyltransferase NSUN3 has been identified as responsible for the first step of the process of f^5^C formation, namely, the methylation of carbon 5 to form methylcytosine (m^5^C). NSUN3 belongs to the family of NOL1/NOP2/Sun (NSUN) domain-containing proteins. Other members of this family of putative RNA methyltransferases have been shown to methylate cytosolic tRNA (NSUN2 and NSUN6) [37,38], cytosolic rRNA (NSUN1/NOP2, NSUN5) [39,40] or mitochondrial rRNA (NSUN4) [41,42].

A large-scale proteomic approach had previously suggested that NSUN3 localizes to the mitochondrial matrix [43]. Having confirmed the mitochondrial localization of the NSUN3 protein, a number of high-throughput techniques employed by different groups further identified mt-tRNA^Met^ as the target of NSUN3. Firstly, ultraviolet crosslinking and immunoprecipitation coupled with high-throughput sequencing (HITS-CLIP), identified mt-RNA^Met^ by irreversibly binding the protein to its target RNA [34,35]. Likewise, methylation-individual nucleotide resolution cross-linking and immunoprecipitation (miCLIP), which relies on the overexpression of a mutated protein that irreversibly binds to the methylation site, arrived at the same conclusion. The same is true for exposure to the cytidine derivative 5-Azacytidine (5-AzaC), which becomes incorporated into nascent RNA and specifically traps m^5^C RNA methyltransferases on their target in 5-azacytidine cross-linking and analysis of cDNA (5-AzaC CRAC) [35].

The above mentioned three studies which identified NSUN3 as the first step enzyme towards f^5^C formation have used different approaches to study the consequences of its inactivation, namely CRISPR-Cas9 generated knockout human embryonic kidney (HEK293T) cells [33], patient derived primary dermal fibroblasts that carry compound heterozygous predicted loss-of-function variants in NSUN3 [34] and small interfering RNA (siRNA) treated HeLa cells [35], and yet reached generally similar conclusions. The lack of NSUN3 in human cells results in the loss of m^5^C34 and f^5^C34 of mt-tRNA^Met^. Furthermore, in vitro reconstitution experiments in combination with mass spectrometry also prove that NSUN3 is required for methylation of mt-tRNA^Met^ [33].

**Figure 2:**
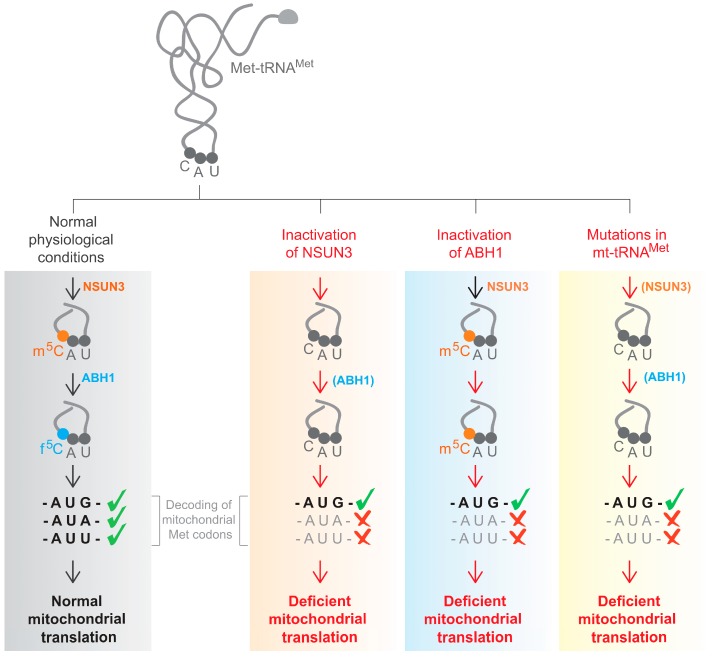
Formation of f^5^C by NSUN3 and ABH1 is crucial for codon recognition and normal mitochondrial translation (grey). Inactivation of NSUN3 (orange) or ABH1 (blue) abolishes the formation of f^5^C34 in mt-tRNA^Met^. Also, mutations in mitochondrial DNA that affect mt-tRNA^Met^ can lead to perturbations in the biogenesis of f^5^C34 (yellow). Proteins in brackets are available, but cannot perform their function because the correct substrate is missing. All three scenarios result in a failure of codon recognition, causing a mitochondrial translation deficiency.

The enzyme responsible for the further conversion of 5-methylcytosine to 5-formylcytosine was identified as ABH1 (ALKBH1), a member of the AlkB-like Fe^2+^/α-ketoglutarate-dependent dioxygenases [35]. Other members of this family have been shown to play a role in DNA repair by removal of alkyl adducts from nucleobases by oxidative dealkylation [44]. Depletion of ABH1 abolishes the formation of f^5^C34 in mt-tRNA^Met^ [35] (Figure 2). Hydroxymethylcytosine (hm^5^C) was not observed as an intermediate in vitro. Although the presence of this modification cannot be ruled out, results seem to indicate that hm^5^C might not play an important role for mt-tRNA^Met^.

Formylation of m^5^C34 of mt-tRNA^Met^ is not the only function of ABH1 reported thus far. It also mediates the demethylation of *N*^1^-methyladenosine in tRNAs and can modulate translation initiation and elongation by regulating the cellular levels of initiator tRNA^iMet^ in the cytoplasm [45]. ABH1 deficiency in mice results in an 80% reduction of the litter size due to embryonic lethality, with the surviving mice exhibiting neural development defects and sex-ratio distortion [46,47]. Notably, incubation of total tRNA with ABH1 led to a significant decrease in the *N*^1^-methyladenosine (m^1^A) level, but not levels of m^5^C [45]. Finally, ABH1 has also been shown to demethylate *N*^6^-methyladenine, preferably on single stranded DNA, suggesting that the demethylation may be coupled with cellular transcription or replication [48].

## 3. The Role f^5^C34 in mt-tRNA^Met^

As introduced earlier, owing to the specific features of the mammalian mitochondrial genetic code, the single tRNA^Met^ bearing a CAU anticodon recognizes the conventional methionine AUG codon as well as the AUA and AUU codons, conventionally coding for isoleucine.

Analysis of the chemically synthesised, f^5^C34-modified anticodon loop of human mitochondrial tRNA^Met^ showed that f^5^C34 contributes to the mt-tRNA’s anticodon domain structure [49]. It was further demonstrated that f^5^C34 defined a reduced conformational space for the nucleoside due to a reduction in conformational dynamics of the anticodon bases [50]. The modification enhances the thermodynamic properties of the anticodon and its ability to bind the unconventional methionine codon AUA. Further analysis revealed that f^5^C is particularly important for AUA recognition at the ribosomal A-site and affects the kinetics of codon recognition at both the P- and A-sites [50]. Visualization of the codon–anticodon complex by X–ray crystallography showed that recognition of both G and A at the third position of the codon occurs in the canonical Watson–Crick geometry [51]. The f^5^C modification shifts the tautomeric equilibrium toward the rare imino-oxo tautomer of cytidine making base pairing with A possible. For more information on the structural insights into f^5^C in an RNA duplex, we refer to Wang et al. [52].

It is currently still undefined whether in vivo, the entire pool of mt-tRNA^Met^ has the f^5^C34 modification or whether differentially modified forms are used to modulate mitochondrial translation activity. In vitro codon recognition studies with chemically synthesised modified or unmodified mt-tRNA^Met^, have demonstrated that during initiation, both the AUG and AUA codon in the ribosomal P-site were preferentially recognized by m^5^C34-modified mt-tRNA^Met^ [35]. Binding of f^5^C modified mt-tRNA^Met^ to these two codons was much lower and not significantly different compared to unmodified C34 mt-tRNA^Met^. Recognition of AUU was generally lower and not significantly different with respect to the mt-tRNA^Met^ modification. Notably, m^5^C34-modified mt-tRNA^Met^ was less efficient than other variants in AUG decoding during elongation. This suggests that m^5^C34 plays an actual role in mitochondrial translation, rather than just being an intermediate step in the f^5^C34 formation. The presence of substantial levels of m^5^C34 in mt-tRNA^Met^ was also detected by next generation sequencing methods derived from RNA bisulfite sequencing (BS). RNA BS is a well-established method to detect m^5^C and hm^5^C. However, since bisulfite does convert f^5^C, this approach cannot distinguish f^5^C from unmodified C. Reduced bisulfite RNA sequencing (Red BS RNA-Seq) relies on the chemical reduction of f^5^C to hm^5^C by NaBH_4_, with the resulting hm^5^C being subsequently detected by RNA BS. Alternatively, 5-formylcytosine chemically assisted bisulfite RNA sequencing (fCAB RNA-Seq), is based upon *O*-ethylhydroxylamine protection of f^5^C from bisulfite conversion. These two approaches measured about 38% f^5^C, 30% m^5^C (and hm^5^C) and 32% unmodified mt-tRNA^Met^ C34 [34]. It is possible, however, that these approaches overestimate the levels of unmodified C due to an inefficient conversion/protection of f^5^C. It should also be noted that this technique cannot distinguish mature mt-tRNA^Met^ from precursors.

The functional in vitro codon recognition studies and the results of the genome-wide detection of f^5^C, however, are in disagreement with the mass spectrometry analysis shown by Nakano et al., 2016 [33], which suggests that the entire pool of mt-tRNA^Met^ has the f^5^C34 modification with undetectable amounts of m^5^C34 or unmodified C34. Therefore, further study will be required to establish whether alterations to the relative abundance of m^5^C34 and f^5^C34 modifications could participate in the regulation of mitochondrial translation.

There is currently no evidence that f^5^C34 is involved in mt-RNA^Met^ aminoacylation. Methionyl-tRNA synthetase (MetRS, MARS2) recognizes mt-tRNA^Met^ irrespective of the presence or absence of f^5^C34 without influencing the kinetics of aminoacylation [53]. This is supported by high-resolution Northern blot analysis on patient fibroblasts lacking a functional NSUN3 protein, and consequently lacking any C34 modification of mt-tRNA^Met^, showing no differences in aminoacylation levels compared to control fibroblasts [34].

Although current evidence supports a role for f^5^C in recognition of both the AUG and AUA codon in both the ribosomal A- and P-site, the exact function has yet to be elucidated. Nonetheless, severe impairment of de novo mitochondrial translation, with a consequent defect in oxygen consumption rate, was consistently observed upon inactivation of NSUN3 or ABH1 [33,34,35]. Therefore, the analysis of cells with the deficiency of NSUN3 or ABH1 has provided the first evidence for a physiological role of f^5^C34 in mt-tRNA^Met^ in living cells.

## 4. The Role of f^5^C34 in mt-tRNA^Met^ in Human Disease

Mitochondria contain multiple genomes per cell. As a result, mtDNA mutations may be present at any fraction, a condition referred to as heteroplasmy. The percentage of mutant mtDNA may vary among patients and among organs and tissues within the same individual. This partially explains the varied clinical phenotype seen in individuals with pathogenic mtDNA mutations. Different base substitutions in the same mt-tRNA or even the same point mutation can cause different clinical symptoms. Despite only accounting for approximately 5% of the total mtDNA sequence, pathogenic point mutations in mt-tRNAs are responsible for the majority of mitochondrial DNA diseases [54,55]. Some of these pathogenetic alterations have been shown to interfere with post-transcriptional mt-tRNA modifications [18,56,57]. The effects of primary mt-tRNA mutations on maturation and post-transcriptional modifications are discussed elsewhere [1,58,59].

Eight pathogenic mutations in mt-tRNA^Met^ have been reported to date with a broad range of symptoms (MITOMAP) [60]. While m.4335A > G is associated with maternally inherited hypertension or Leber’s hereditary optic neuropathy [61,62], m.T4409T > C and m.G4450G > A cause myopathy [63,64] and m.4437C > T is associated with hypotonia, seizures, muscle weakness, lactic acidosis and hearing loss [65]. Differentially affected levels of C34 modifications in mt-tRNA^Met^ could provide a possible explanation for this wide clinical phenotypic variation in the symptoms associated with mutations in the same mt-tRNA. Two out of eight mutations (m.A4435A > G and m.C4437C > T) inhibited NSUN3-mediated m^5^C formation in vitro, suggesting that in these mutations, the molecular pathogenesis can be at least partially attributed to the absence of f^5^C in mt-tRNA^Met^ [33]. Both these mutations are localized in the anticodon arm of the mt-tRNA^Met^. These results are in line with another study that shows that NSUN3 requires a stable anticodon stem loop for methylation of cytosine 34 [35]. These in vitro results indicate that some, but not all, mutations in mt-tRNA^Met^ cause hypomethylation, resulting in a reduced level of f^5^C.

The importance of this post-transcriptional modification of C34 of mt-tRNA^Met^ is further supported by our recent study that describes a patient who has no detectable levels of m^5^C34 or f^5^C34. Whole-exome sequencing had identified compound heterozygous predicted loss-of-function variants in the *NSUN3* gene and no functional NSUN3 protein was detected. This patient developed mitochondrial disease symptoms at the age of three months. Symptoms were combined developmental disability, microcephaly, failure to thrive, recurrent increased lactate levels in plasma, muscular weakness, external ophthalmoplegia and convergence nystagmus [34]. Taken together, these data show that detailed understanding of the basic mechanistic aspects of mt-tRNA modifications might be helpful for explaining the different clinical presentations of mitochondrial diseases related to genetic defects in the molecular biology of mt-tRNA.

## 5. Concluding Remarks

Methylation of C34 in mt-tRNA^Met^ by NSUN3, followed by further oxidation to f^5^C34 by ABH1, is essential for mitochondrial translation and mitochondrial function. However, it is still unclear whether m^5^C34 in mt-tRNA^Met^ is a transient intermediate or whether this modification plays an actual role in mitochondrial translation regulation. Furthermore, it would also be interesting to check for additional functions of ABH1 in mitochondria that could contribute to tuning translation initiation and elongation. To summarize, although current evidence supports an important role for f^5^C34 and possibly m^5^C34 in codon recognition in both initiation and elongation, the exact in vivo role has yet to be elucidated.
